# Dynamic alterations in PD-1/PD-L1 expression level and immune cell profiles based on radiation response status in mouse tumor model

**DOI:** 10.3389/fonc.2022.989190

**Published:** 2022-11-21

**Authors:** Yi Na Yoon, Min Ho Choe, Moonkyoo Kong, Weon Kuu Chung, Jae-Sung Kim, Yu Jin Lim

**Affiliations:** ^1^ Division of Radiation Biomedical Research, Korea Institute of Radiological and Medical Sciences, Seoul, South Korea; ^2^ Department of Radiation Oncology, Kyung Hee University Medical Center, Kyung Hee University College of Medicine, Seoul, South Korea; ^3^ Department of Radiation Oncology, Kyung Hee University Hospital at Gangdong, Kyung Hee University College of Medicine, Seoul, South Korea; ^4^ Radiological and Medico-Oncological Sciences, University of Science and Technology, Daejeon, South Korea

**Keywords:** radiotherapy, PD-1/PD-L1, dynamic alterations, dose fractionation, tumor immune microenvironment, flow cytometry, transcriptome

## Abstract

**Introduction:**

Based on the immunologic effects of anti-cancer treatment and their therapeutic implications, we evaluated radiotherapy (RT)-induced dynamic alterations in programmed death-1 (PD-1)/PD ligand-1 (PD-L1) expression profiles.

**Methods:**

Local RT with 2 Gy × 5 or 7.5 Gy × 1 was administered to the CT26 mouse model. Thereafter, tumors were resected and evaluated at the following predefined timepoints according to radiation response status: baseline, early (immediately after RT), middle (beginning of tumor shrinkage), late (stable status with RT effect), and progression (tumor regrowth). PD-1/PD-L1 activity and related immune cell profiles were quantitatively assessed.

**Results:**

RT upregulated PD-L1 expression in tumor cells from the middle to late phase; however, the levels subsequently decreased to levels comparable to baseline in the progression phase. RT with 2 Gy × 5 induced a higher frequency of PD-L1+ myeloid-derived suppressor cells, with a lesser degree of tumor regression, compared to 7.5 Gy. The proportion of PD-1+ and interferon (IFN)-γ+CD8α T cells continued to increase. The frequency of splenic PD-1+CD8+ T cells was markedly elevated, and was sustained longer with 2 Gy × 5. Based on the transcriptomic data, RT stimulated the transcription of immune-related genes, leading to sequentially altered patterns.

**Discussion:**

The dynamic alterations in PD-1/PD-L1 expression level were observed according to the time phases of tumor regression. This study suggests the influence of tumor cell killing and radiation dosing strategy on the tumor immune microenvironment.

## Introduction

Radiotherapy (RT) is a major component of conventional anticancer therapies. Owing to its potent tumor cell-killing effect, RT is used to treat more than two-thirds of patients with cancer (1). As clinical interest in cancer immunotherapy has increased over the past decade, RT has recently been revealed to be a double-edged sword that elicits both immune-stimulatory and inhibitory effects in the tumor microenvironment (2). Local RT enhances antigen presentation with increased DNA damage, leading to the upregulation of pro-inflammatory cytokine levels and cytotoxic T cell-mediated immunogenic cell death. RT can also mediate immunosuppressive responses with increased recruitment and infiltration of key components, such as regulatory T cells (Tregs), myeloid-derived suppressor cells (MDSCs), and immune checkpoint-related molecules (3). The dynamic alterations in immune cell profiles are essential for boosting the tumor-targeting immune responses with RT. However, in real-world clinical settings, the longitudinal assessment of immunomodulatory reactions is not feasible due to the need for invasive procedures.

The programmed death-1 (PD-1)/programmed death ligand-1 (PD-L1) axis is an immune checkpoint, and an important target that is extensively used in cancer immunotherapy. In recent years, several *in vivo* studies have revealed the synergistic tumoricidal effects of combining RT with α-PD-1/PD-L1 inhibitors (4–6). Based on laboratory data, several clinical trials sought to expand the clinical application of radioimmunotherapy, especially for advanced or metastatic tumors (7–10). However, the clinical benefits of the combinatory treatment in humans have not been as high as expected from the mouse model (11). To date, there is no clear consensus on the optimal timing or dose-fractionation strategy for combining RT with immunotherapeutic drugs.

In the present study, we hypothesized that the RT-induced immunologic impact on the major target for immunotherapy, the PD-1/PD-L1 axis, is not stationary, but dynamic in nature. To understand the synergistic effects of combining RT with the PD-1/PD-L1 blockade, dynamic RT-induced alterations in tumor immune status and blood-borne biomarkers in relation to PD-1/PD-L1 expression must be explored. To assess the predefined post-RT response status, sequential alterations in the PD-1/PD-L1 expression profiles and relevant immune cell compositions were quantitatively assessed using a CT26-based *in vivo* model. This study would provide additional data regarding the impact of RT on the tumor immune microenvironment.

## Materials and methods

### Mouse tumor model

Six-week-old female BALB/c mice obtained from Orient Bio Inc. (Gyeonggi-do, Republic of Korea) were used in this study. Mice were maintained in specific pathogen-free facilities. The animal experimental protocol was approved by the Institutional Animal Care and Use Committee of the Korea Institute of Radiological and Medical Sciences (Seoul, Republic of Korea) (approval numbers: kirams2019-0026, kirams2020-0064). All experimental procedures were conducted in accordance with the ethical regulations and standards of the Korea Institute of Radiological and Medical Sciences (Seoul, Republic of Korea). The CT26 cells were purchased from the American Type Culture Collection (ATCC, VA, USA). The cells were grown in an incubator in a humidified atmosphere of 95% air and 5% CO_2_ at 37.5°C. CT26 cells were maintained in Dulbecco’s modified Eagle’s medium supplemented with 10% fetal bovine serum (35-015-CVR; Corning, NY, USA) and 1% penicillin/streptomycin (30-002-CI; Corning). A total of 2 × 10^5^ CT26 cells in 50 μL of Dulbecco’s phosphate-buffered saline were subcutaneously injected into the right flank of mice. The perpendicular diameters (width/length) of the tumors were measured every 2 or 3 days, and the individual tumor volume was calculated using the formula: 1/2 × length × width^2^ (mm^3^). Palpable murine tumors (< 150 mm^3^) were selected and precisely irradiated with 200 kVp X-rays using a self-contained x-ray system X-RAD 320 (Precision X-Ray Inc., CT, USA). Each mouse was anesthetized and placed on a fixed shelf with an illuminator to expose the tumor site and prevent any radiation effects on normal organs at risk. The irradiation field for each mouse was confined to the tumor site with a highly homogenous beam, which indicates local RT throughout this study.

### Flow cytometry

Tumor and spleen tissues were cut into small pieces and minced using scalpels. For tumor dissociation, the sample was soaked in media containing cocktail enzymes (Tumor dissociation kit; Miltenyi Biotec, Bergisch Gladbach, Germany), according to the manufacturer’s recommendations. Spleen tissues were homogenized and filtered through a 70 μm nylon cell strainer, and 1× RBC lysis buffer (Sigma-Aldrich, MO, USA) was added to eliminate the red blood cells. Single cells were initially stained with a Fixable Viability Stain 780 (BD, NJ, USA) for 15 min at room temperature (25–27°C) and then incubated with anti-CD16/32 antibodies (Thermo Fischer Scientific, MA, USA) for 15 min at room temperature. To isolate intratumoral and splenic CD45^+^ cells, an EasySep™ Mouse CD45^+^ Cell Isolation Kit (StemCell Technologies, Vancouver, Canada) was used, according to the manufacturer’s instructions. Single cell samples of tumor tissues were resuspended in phosphate-buffered saline with 5% fetal bovine serum and 1 mM ethylenediaminetetraacetic acid (fluorescence-activated cell sorting [FACS] buffer) for magnetic labeling. Cells were incubated for 8 min at room temperature with a CD45-positive selection cocktail and tetrameric antibody complexes. Tubes containing the mixture were placed in an EasySep™ magnet for 5 min, and the supernatant was discarded. Finally, magnetically labeled CD45^+^ cells were resuspended in FACS buffer. CD45-negative or -positive samples were stained with monoclonal antibodies (**Table S1**). All events were analyzed using a CytoFLEX flow cytometer (Beckman Coulter, CA, USA). Kaluza Analysis version 2.1.1 (Beckman Coulter) and FlowJo version 10.5.3 (BD) software were used to analyze the flow cytometric data. The representative gating strategies for overall analyses are represented in **Table S2**; **Figures S1**, **S2**.

### Intracellular staining

To carry out simultaneous analysis of interferon (IFN)-γ, single cell suspension samples were stimulated with 1 μM phorbol 12-myristate 13-acetate (Sigma-Aldrich, MO, USA) and ionomycin (Sigma) at 37 °C for 2 h. The Forkhead box P3/transcription factor staining buffer set (Thermo Fischer Scientific) was used to fix and permeabilize the cells, according to the manufacturer’s recommendations. The cells were stained with monoclonal antibodies for 30 min on ice (**Table S2; Figures S1, S2**).

### NanoString analysis

Using the MasterPure™ Complete RNA Purification Kit (Lucigen, MA, USA), RNA was isolated from the sorted intratumoral CD45^+^ cells. NanoString analysis was performed using an nCounter Analysis System (NanoString Technologies, WA, USA), and the nCounter Mouse Immunology Kit (NanoString, WA, USA), which contains 561 immunology-related genes found in mice. Fold-change values for each time phase were calculated based on the gene expression at baseline (n = 3 mice per group).

### Statistical analyses

A two-tailed Student’s t-test was performed to compare the expression levels of immune cell markers at different time points. One-way analysis of variance with Tukey’s *post-hoc* test was used to compare multiple elements. *P*-values less than 0.05 were considered to indicate statistical significance. All data are presented as mean ± standard error of the mean. GraphPad Prism version 9.0.0 (GraphPad Software, CA, USA) was used for all statistical analyses and diagrammatic data presentation.

## Results

### Definition of sequential time points in response to RT

**Figure 1:**
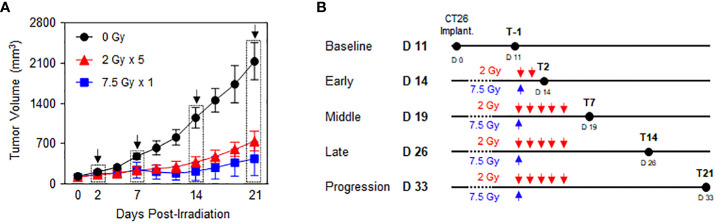
Time-course response phases following RT. CT26 cells were implanted in the right flank of six-week-old female BALB/c mice. After 12 d, the established tumors were locally irradiated with 2 Gy × 5 and 7.5 Gy × 1 (n = 3–4 mice per group). **(A)** Comparison of the tumor growth delay curves of mice irradiated with 0 Gy, 2 Gy × 5, and 7.5 Gy × 1: time points of the surgical evaluation are indicated by dotted rectangles with arrows. **(B)** Details of the radiation dose regimens and post-radiation time points of the surgical evaluation. These figures are based on the representative data obtained from at least two independent experiments. Abbreviation: RT, radiotherapy.

Preliminary experiments were performed to explore the time-course phases in response to RT with fractionated 2 Gy × 5 and 7.5 Gy × 1 according to the comparable biologically effective dose (BED) for tumor cells. Based on the tumor growth delay curves after RT (**Figure 1A**), the following five time phases were determined to assess the dynamic alterations in PD-1/PD-L1 expression level and relevant immune cell compositions in the tumor microenvironment and spleen: baseline (non-irradiated status prior to RT), early (initial status immediately after the initiation of RT), middle (time period of RT effect with tumor shrinkage), late (stable status with maintained RT effect), and progression (delayed tumor regrowth observed with limited RT effect) (**Figure 1B**). By defining day 0 as the initiation of RT, the post-treatment time points of the early, middle, late, and progression were days 2 (T2), 7 (T7), 14 (T14), and 21 (T21), respectively. During the post-RT time course, RT with 2 Gy × 5 resulted in tumor volume regression to a lesser extent than the 7.5 Gy × 1 regimen (**Figure 1A**).

### Upregulated PD-L1 expression levels on tumor cells and intratumoral immune cells with sequential alterations

**Figure 2:**
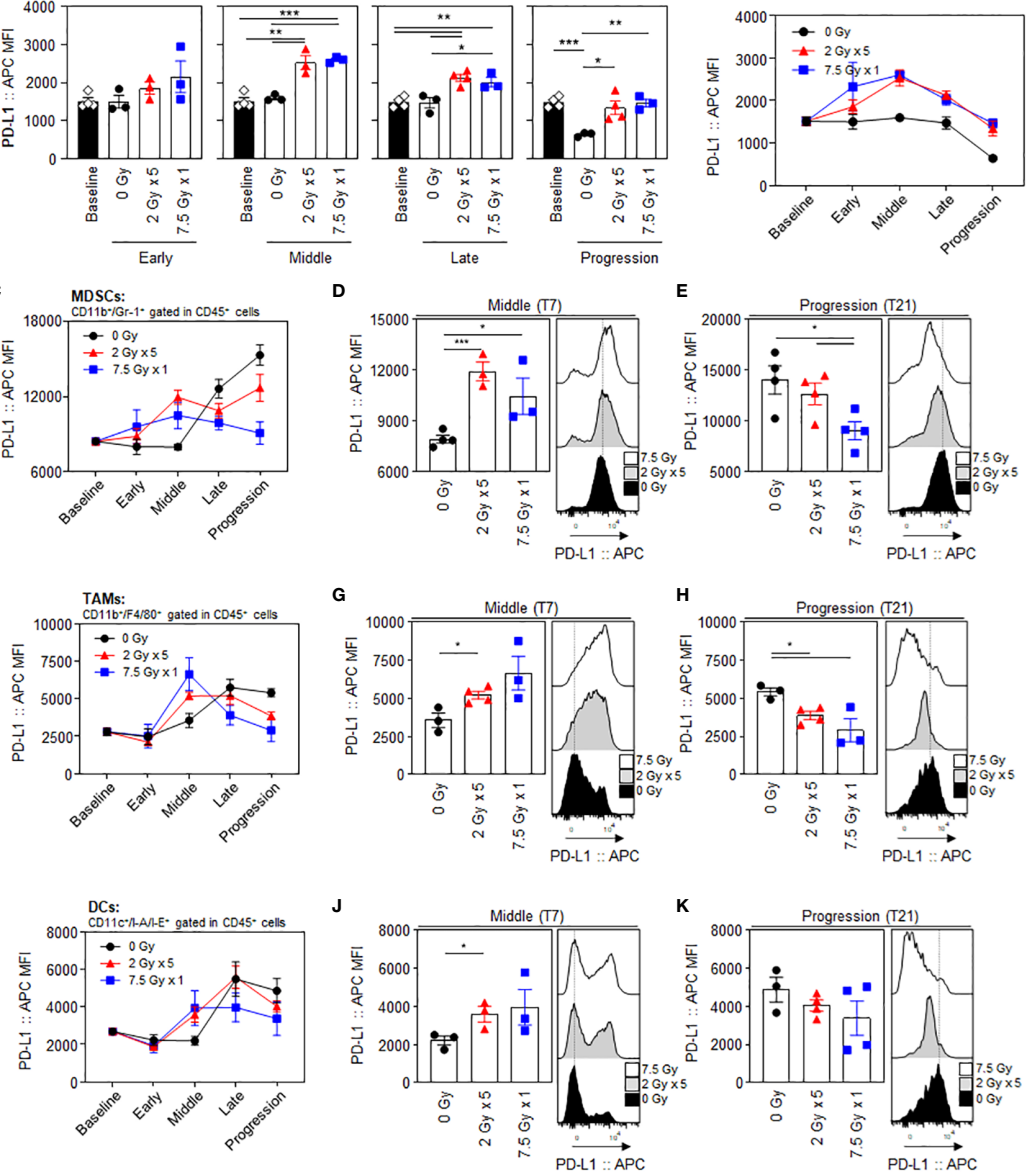
Radiation-induced increase and dynamic alterations in PD-L1 expression levels in tumor cells and intratumoral MDSCs, TAMs, and DCs. Sequential alterations in the PD-L1 MFI values of tumor cells depicted using **(A)** individual bar and **(B)** line graphs. PD-L1 MFI values in MDSCs depicted using **(C)** line graphs, and a comparison of the experimental groups at the **(D)** middle (T7) and **(E)** progression (T21) phases: black dashed lines indicate the geometric mean values of 0 Gy at each time point. PD-L1 MFI values of **(F–H)** TAMs and **(I–K)** DCs: **(F, I)** line graphs and comparisons of the experimental groups at the **(G, J)** middle (T7) and **(H, K)** progression (T21) phases. All data are presented as mean ± SEM. ^*^ *P* < 0.05, ^**^ *P* < 0.01, and ^***^ *P* < 0.001 based on unpaired two-tailed *t*-test. Results of one-way ANOVA analyses for each time phase are listed in **Table S4**. These figures are based on the representative data obtained from at least two independent experiments. The histograms of **(D, E)**, **(G, H)**, and **(J, K)** are from representative individual samples. Abbreviations: PD-L1, programmed death ligand-1; MDSCs, myeloid-derived suppressor cells; TAMs, tumor-associated macrophages; DCs, dendritic cells; MFI, mean fluorescence intensity; SEM, standard error of the mean; ANOVA, analysis of variance.

PD-L1 expression levels in CT26 tumor cells, gated with a CD45-negative population, showed an increasing tendency following RT, especially during the middle (T7) to late (T14) phases. (**Figure 2A**). The mean fluorescence intensity (MFI) levels of PD-L1 in the 2 Gy × 5 and 7.5 Gy × 1 groups were significantly elevated from the middle (T7) phase (*P* = 0.003 and < 0.001 for 2 Gy × 5 and 7.5 Gy × 1 vs. baseline, respectively). In the progression (T21) phase, the PD-L1 levels declined again, and were comparable to those at baseline (**Figure 2B**). Compared to 0 Gy at this time phase, the PD-L1 level of irradiated mice was higher, suggesting that the RT effect remained. RT increased the PD-L1 expression levels during the middle (T7) phase in intratumoral MDSCs (**Figures 2C–E**), tumor-associated macrophages (TAMs) (**Figures 2F–H**), and dendritic cells (DCs) (**Figures 2I–K**). In MDSCs, the PD-L1 levels had a greater increase with 2 Gy × 5 than 7.5 Gy × 1 (*P* < 0.001 and *P* = 0.042 for 2 Gy × 5 and 7.5 Gy × 1 vs. 0 Gy, respectively), and the tendency was maintained until the progression (T21) phase (*P* = 0.041 for 2 Gy × 5 vs. 7.5 Gy × 1). The expression level was highest in non-irradiated mice (0 Gy) at the end of the observation, which also supports the limited RT effect in the 2 Gy × 5 group.

### Elevated PD-1 expression level on tumor-infiltrating CD8α^+^ T cells and its maintenance until tumor progression

**Figure 3:**
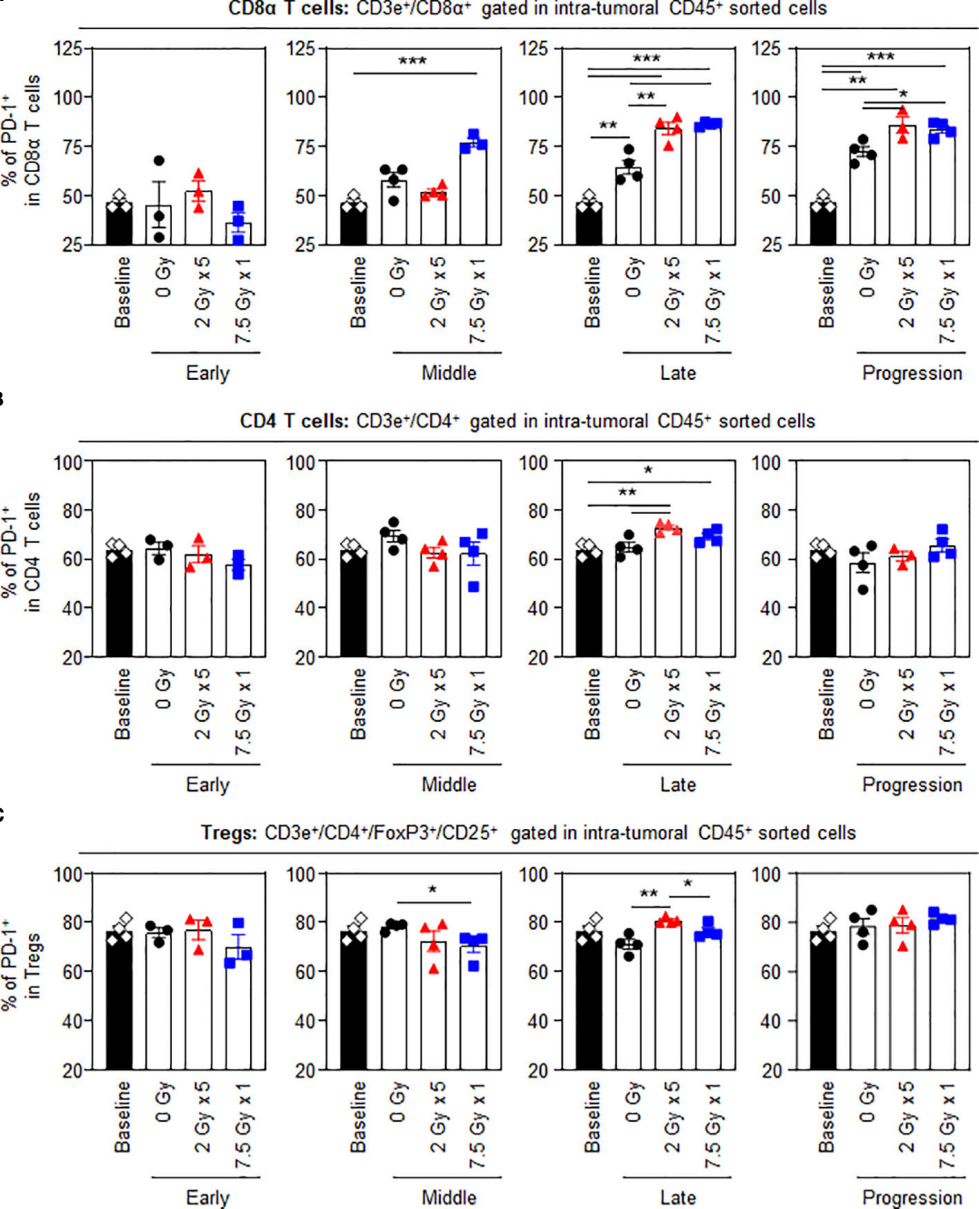
PD-1 levels in intratumoral T-cell subsets following RT. Altered proportions of intratumoral **(A)** PD-1^+^CD8α T, **(B)** PD-1^+^CD4 T, and **(C)** PD-1^+^ Treg cells for each time phase. All data are presented as mean ± SEM. ^*^ *P* < 0.05, ^**^ *P* < 0.01, and ^***^ *P* < 0.001 based on unpaired two-tailed *t*-test. Results of one-way ANOVA analyses for each time phase are listed in **Table S4**. These figures are based on the representative data obtained from at least two independent experiments. Abbreviations: PD-1, programmed death-1; RT, radiotherapy; Treg, regulatory T; SEM, standard error of the mean; ANOVA, analysis of variance.

Unlike other T cell subsets, intratumoral CD8α^+^ T cells displayed dynamic changes in the proportion of PD-1^+^ cells following RT. Treatment with 7.5 Gy × 1 caused an abrupt increase in the expression levels of PD-1 in CD8α^+^ T cells in the middle (T7) phase (*P* < 0.001 for 7.5 Gy × 1 vs. baseline). However, the effect with 2 Gy × 5 was initiated in the late (T14) phase (*P* < 0.001 for both comparisons of 2 Gy × 5 and 7.5 Gy × 1 vs. baseline): the RT-induced elevation in PD-1 level was also commonly observed as compared to the 0 Gy group (*P* = 0.006 and 0.001 for comparisons of 2 Gy × 5 and 7.5 Gy × 1 vs. baseline, respectively). The elevated PD-1^+^CD8α T-cell population in the irradiated mice was persistently maintained until the last progression (T21) phase, which was valid in both comparisons with the baseline (*P* = 0.001 and < 0.001 for 2 Gy × 5 and 7.5 Gy × 1, respectively) and 0 Gy (*P* = 0.033 and 0.012 for 2 Gy × 5 and 7.5 Gy × 1, respectively) (**Figure 3A**). Meanwhile, the expression levels of PD-1 in CD4 T cells and Tregs were moderately increased with 2 Gy × 5 at the late (T14) phase (**Figures 3B, C**) compared to levels in mice administered 0 Gy.

### Effects of local RT on PD-1 expression level on splenic T cells

**Figure 4:**
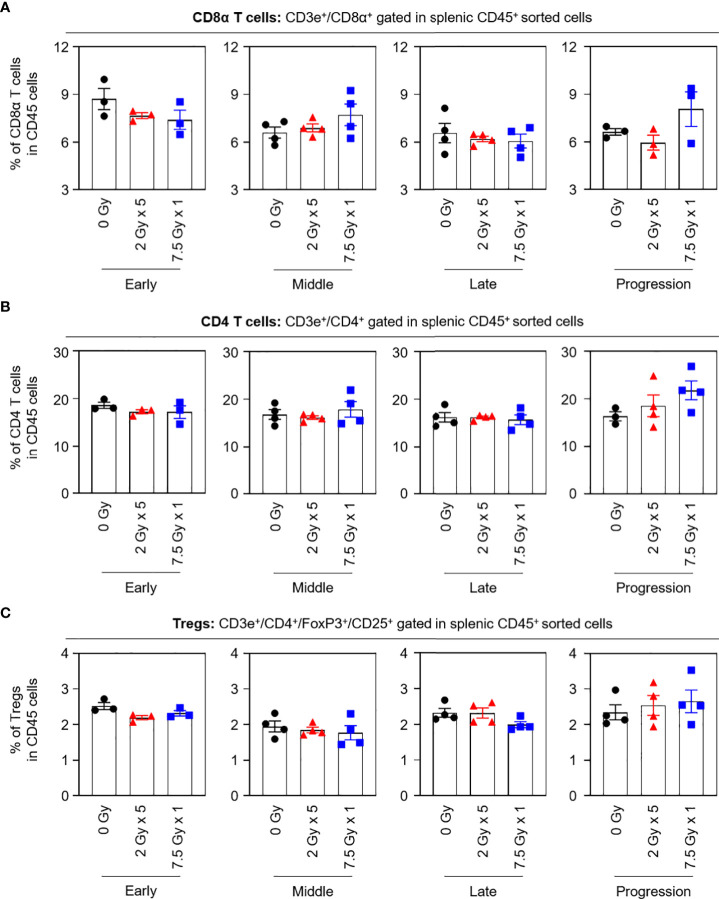
Compositions of the splenic T-cell subsets. Proportions of **(A)** CD8α T, **(B)** CD4 T, and **(C)** Tregs in each time phase. These figures are based on the representative data obtained from at least two independent experiments. Abbreviation: Tregs, regulatory T cells; ANOVA, analysis of variance.

**Figure 5:**
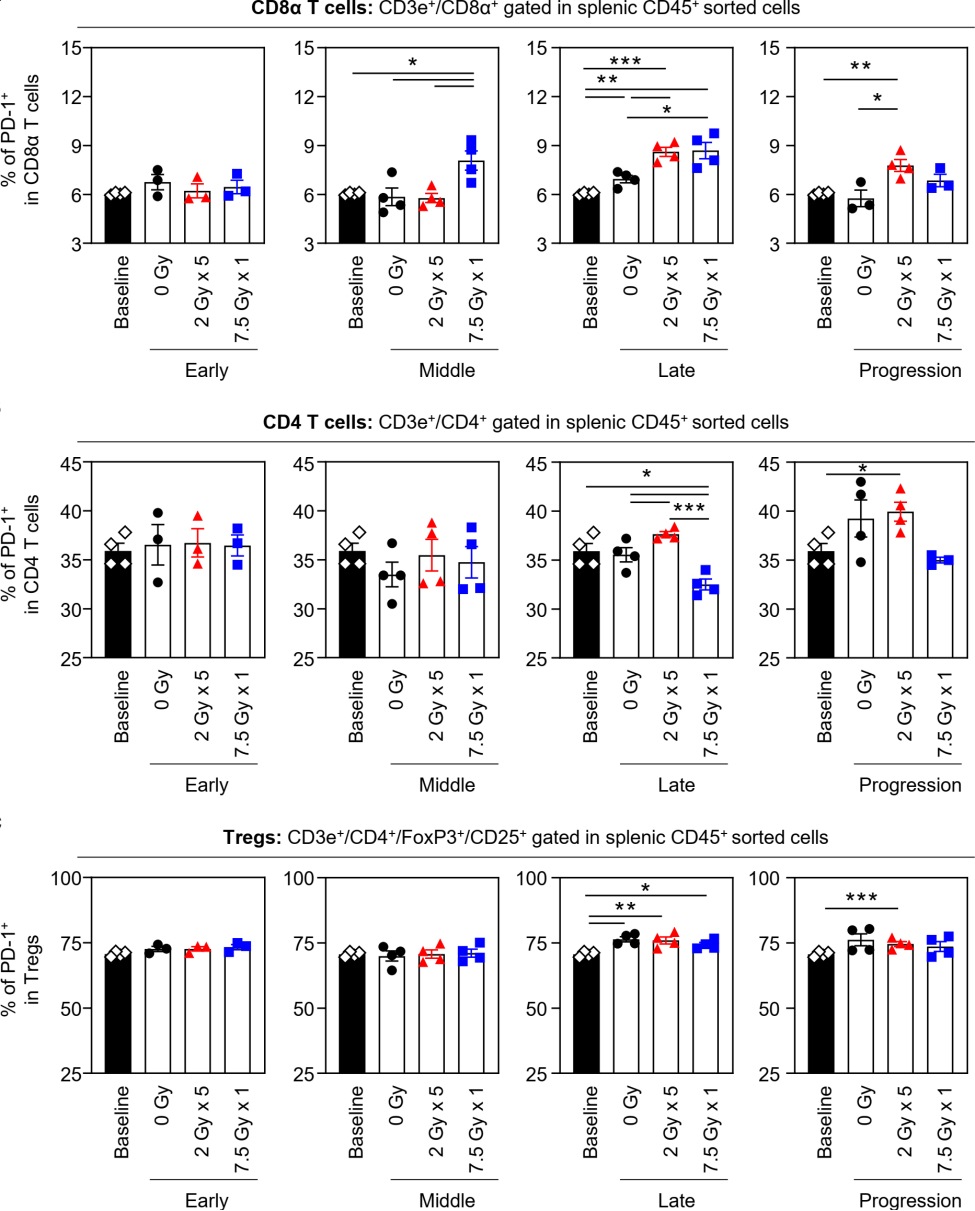
Effects of local RT on splenic PD-1^+^ T-cell subsets. Altered proportions of splenic **(A)** PD-1^+^CD8α T, **(B)** PD-1^+^CD4 T, and **(C)** PD-1^+^ Treg cells for each time phase. All data are presented as mean ± SEM. ^*^ *P* < 0.05, ^**^ *P* < 0.01, and ^***^ *P* < 0.001 based on unpaired two-tailed *t*-test. Results of one-way ANOVA analyses for each time phase are listed in **Table S4**. These figures are based on the representative data obtained from at least two independent experiments. Abbreviations: RT, radiotherapy; PD-1, programmed death-1; Treg, regulatory T; SEM, standard error of the mean; ANOVA, analysis of variance.

When the potential systemic antitumor responses following RT were evaluated, the proportions of splenic T-cell subsets were not found to significantly change after local RT (**Figure 4**). However, the frequency of splenic PD-1^+^CD8^+^ T cells was increased by 7.5 Gy × 1 and 2 Gy × 5 in the middle (T7) and late (T14) phases, respectively, which was valid in both comparisons with the baseline and 0 Gy. In particular, in the 2 Gy × 5 group, the increased pattern continued to the progression (T21) phase (**Figure 5A**). The proportions of PD-1^+^CD4^+^ T and PD-1^+^ Treg cells were also elevated with 2 Gy × 5 over the late (T14) and progression (T21) phases, which did not occur with 7.5 Gy × 1 (**Figures 5B, C**).

### RT-induced alterations in the proportion of IFN-γ^+^CD8α T cells and median fluorescence intensity

**Figure 6:**
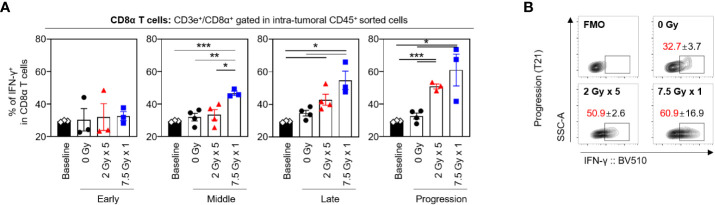
Sequential changes in the population of the intratumoral effector CD8α T cells after RT. **(A)** Altered proportions of IFN-γ^+^CD8α T cells at each time phase. **(B)** Representative contour plot images of the IFN-γ^+^ population at the progression phase. All data are presented as mean ± SEM. ^*^ *P* < 0.05, ^**^ *P* < 0.01, and ^***^ *P* < 0.001 based on unpaired two-tailed *t*-test. Results of one-way ANOVA analyses for each time phase are listed in **Table S4**. These figures are based on the representative data obtained from at least two independent experiments. The contour plots of **(B)** are from representative individual samples. Abbreviations: RT, radiotherapy; IFN, interferon; SEM, standard error of the mean; ANOVA, analysis of variance.

The frequencies of CD8α^+^ T cells secreting IFN-γ tended to increase following RT, and this tendency was maintained until the progression (T21) phase (**Figures 6A, B**). With 7.5 Gy × 1, the proportion of IFN-γ^+^CD8α T cells was found to be elevated from the middle (T7) phase and was maintained over the late (T14) and progression (T21) phases, which was valid in both comparisons with baseline (*P* < 0.001, *P* = 0.011, and 0.033 for T7, T14, and T21, respectively) and 0 Gy (*P* = 0.005, 0.012, and 0.021 for T7, T14, and T21, respectively). However, in mice irradiated with 2 Gy × 5, an alteration was observed in the late (T14) phase.

### Transcriptional status of immune-stimulatory and inhibitory genes depending on the post-RT time phases

**Figure 7:**
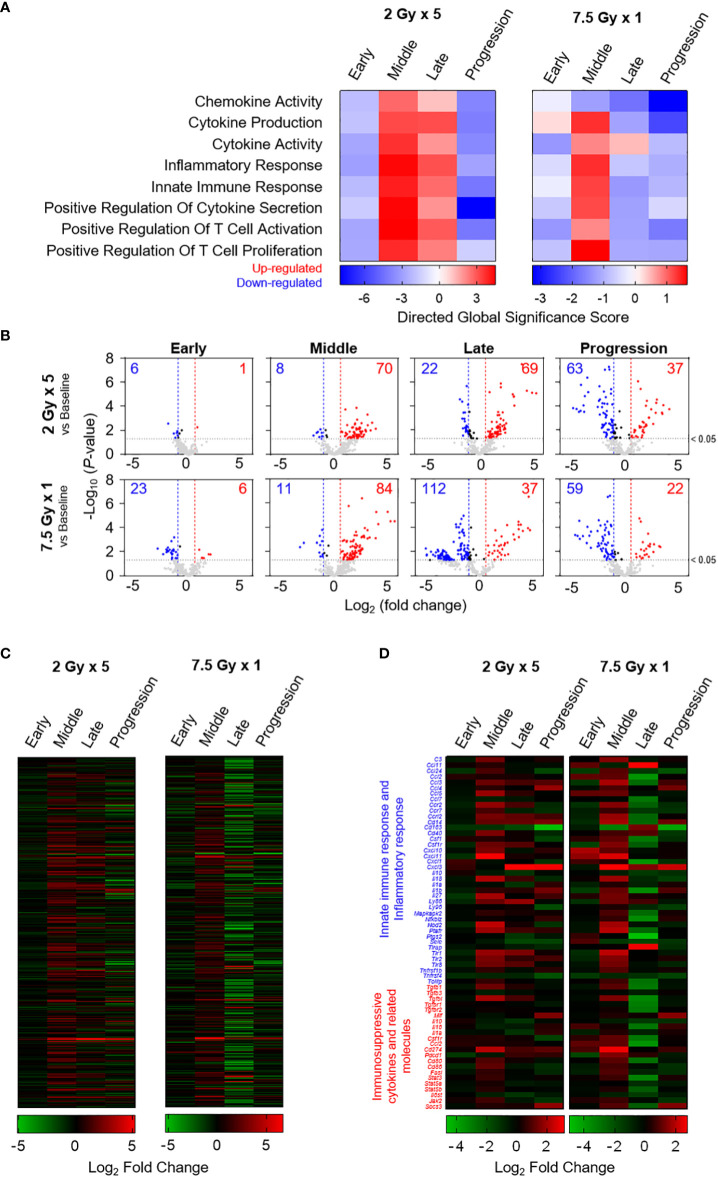
Transcriptomic analyses representing sequential alterations in the transcription levels of immunomodulatory gene sets. **(A)** Directed global significance scores revealing the altered patterns of categorized gene signatures. **(B)** Volcano plots representing the expression levels of transcripts at each time phase as indicated on each panel. Heatmaps are based on the mean Log_2_ fold-change values for the expression levels of **(C)** 382 genes and **(D)** selected genes related to innate immune response, inflammatory response, and immunosuppressive cytokines and related molecules. These figures are based on the representative data obtained from at least two independent experiments.

Based on the directed global significance statistics of the transcriptomic data, the transcription levels of gene sets related to immunomodulation were upregulated in the middle (T7) phase. Unlike the 2 Gy × 5 group, the elevated levels in the 7.5 Gy × 1 group were abruptly downregulated (**Figure 7A**). Similarly, as depicted in the volcano plots of 7.5 Gy × 1, the number of upregulated genes was maximal (n = 84) in the middle (T7) phase; however, many more genes (n = 112) were abruptly downregulated in the late (T14) phase (**Figure 7B**). Heatmap analysis also revealed RT-induced sequential alterations in the overall immune-stimulatory and inhibitory genes, showing sequentially altered patterns (**Figures 7C, D**).

## Discussion

Owing to the impact of conventional cytotoxic therapies on tumor-targeting immune responses, we sought to quantitatively evaluate the dynamic characters of RT-induced alterations in PD-1/PD-L1 expression level and immune cell composition. PD-L1 expression levels in tumor cells and intratumoral MDSCs, TAMs, and DCs were upregulated following RT, mainly during the period of sufficient tumor regression. With tumor regrowth, PD-L1 levels in tumor cells were found to decrease to levels comparable to those at baseline. Compared to 7.5 Gy × 1, RT with 2 Gy × 5, calculated based on a comparable BED level, resulted in a higher frequency of PD-L1^+^ MDSCs with a lesser degree of tumor volume regression. Although local RT to the tumor site was not sufficient to potentiate alterations in splenic proportions of CD8α T, CD4 T, and Treg cells by themselves, the number of splenic PD-1^+^ T cells was significantly elevated and sustained with 2 Gy × 5. Based on the transcriptomic data of gene sets related to immunomodulation, RT stimulated the transcription of genes involved in both immune-stimulatory and inhibitory responses with sequentially altered patterns.

The upregulated PD-1/PD-L1 expression level with RT effects is in line with the results of previous *in vivo* studies (4, 5). However, this study initially sought to evaluate the phenomena according to the course of radiation responses. Immune-stimulating signals triggered by irradiation are well known to enhance T cell infiltration into tumor tissues, and antigen-specific binding of the T-cell receptor (TCR) molecule activates the *Pdcd1* gene (12). Also, TCR signaling induces the release of IFN-γ and various oncogenic signaling pathways, mainly contributing to the induction of PD-L1 expression (13). In our study, the elevated PD-L1 expression levels subsequently decreased after the regrowth, suggesting less potent PD-1/PD-L1 binding activity with tumor progression. Based on our exploratory analysis of the transcriptional status of IFN-γ-related genes, no significant post-RT alterations occurred in either the immunostimulatory or immunosuppressive responses at the end of the evaluation (**Figure S3**). Taken together, we suggest that the tumor-inflamed change *via* irradiation was mainly valid during the response of tumor regression, which was not persistently sustained after the regrowth status.

Interestingly, in the early phase, immediately after the initiation of RT, no significant effect was observed. Accordingly, the time interval of 1–2 days was insufficient for RT-induced alterations in the immunologic tumor microenvironment, which is consistent with the results of Kim et al. (14). For RT-induced immunogenic cell death, direct tumor cell-killing induces a surge in tumor antigen loading, and related cascade reactions consequently upregulate the expression levels of effector T cells and proinflammatory cytokines (15, 16). As the immune cell alterations by RT with 2 Gy × 5 appeared later and lasted longer than a single 7.5 Gy, such results suggest an association between the changeable PD-1/PD-L1 expression level and the dynamic nature of tumor shrinkage. Therefore, in line with the well-known principle regarding RT-induced effects, the tumoricidal effects of ionizing radiation might affect the immunomodulatory responses.

To better understand the differing alterations according to the dosing strategies, we compared fractionated 2 Gy × 5 and single 7.5 Gy based on a linear-quadratic model. Although a similar degree of tumor cell-killing was expected, RT with 2 Gy × 5 resulted in tumor volume regression to a lesser extent throughout the observation period. Regarding the varying degrees of radiotherapeutic efficacy, MDSCs play a crucial role in acquiring resistance to RT (17, 18). Lan et al. recently demonstrated that ablative hypofractionated RT reduced the recruitment of MDSCs and decreased PD-L1 expression levels in MDSCs compared with the conventional fractionated regimen (19). A dose-dependent differential tendency was also maintained until the end of the evaluation. Therefore, this study suggested that the hypofractionated RT regimen with a high daily dose, rather than conventional fractionation, might be less affected by the immunosuppressive function of MDSCs. As the dose regimens explored in this study existed within the range of doses used in real-world clinical settings, our results are expected to be relatively feasible for translation to patients. Indeed, these findings further substantiate the rationale for delivering a stereotactic hypofractionated boost to any residual radioresistant cancer tissue after a normofractionated radiotherapy course (20), as encouraged by some preliminary clinical results (21), including also inoperable stage III lung cancer patients who were incompletely responsive to standard radiochemotherapy (22). However, further investigations are necessary to establish an appropriate dose-fractionation strategy.

The abscopal effect is a systemic antitumor response to tumor lesions outside the local RT field that is unusually observed in clinical practice (23–25). Based on the synergistic pro-inflammatory effects induced by combining RT with α-PD-1/PD-L1 therapies, radioimmunotherapy has been considered as a therapeutic option to obtain the abscopal phenomenon (26–28). In our study, the proportion of splenic T-cell subsets was not markedly altered following RT, suggesting that the local RT effect was insufficient to elicit systemic changes in T cell-mediated immunity. However, the proportion of splenic PD-1^+^ T cells significantly increased from the middle (T7) or late (T14) phase, according to the dose regimens. In particular, RT with 2 Gy × 5 upregulated PD-1 levels in splenic CD8 T, CD4 T, and Treg subsets, and the alterations lasted longer than those with a single 7.5 Gy. Although inconclusive, our data of the 2 Gy × 5 group can be interpreted in line with other prior investigations that demonstrated a more substantial abscopal effect with fractionated regimens (19, 29).

This study had several limitations. In the absence of mechanistic analyses, we could not determine the underlying molecular mechanisms of the present results. However, preclinical observations at the macroscopic scale play a role in translating the mouse model results to humans and predicting clinical results (30). We also recognize that the novelty of this study might be questioned because other previous studies have also tested and reported the radiation effects on PD-1/PD-L1 expression levels (31–33). However, most previous studies have checked radiation-induced changes at one post-treatment time point. In our study, the sequential alterations were consecutively evaluated during and after completion of RT. Beyond the changes observed at one time point, we focused on how long such changes persisted and whether they changed again along the post-treatment timeline. To clarify the therapeutic role of RT in the contemporary era of immunotherapy, additional knowledge of the initiation and sustainability of radiation effects in the tumor-immune microenvironment is needed (34). Therefore, we would like to highlight that this is the first study to evaluate radiation-induced dynamic changes in PD-1/PD-L1-related immune cell profiles. Although each mouse-derived cancer cell line differs in terms of immunogenicity and radiosensitivity (35), we preferentially selected the CT26 cell line, which is radiosensitive and has highly immunogenic profile (36). The data of absolute numbers of tumor-infiltrating leukocytes may also be useful to assess the level of infiltration, but the relative changes in the expression level of PD-1/PD-L1, following RT, were determined as the primary outcome, regarding the scope of this study. Since our data were not additionally verified using other mouse tumor models of radioimmunotherapy, this study cannot be conclusive, but rather hypothesis-generating with support to other previous investigations. Nevertheless, for human cancers, a longitudinal assessment to quantify immune cell composition and immunomodulatory biomarkers within the tumor microenvironment inevitably accompanies invasive surgical procedures. Therefore, tracking tumor immune status during and after cytotoxic therapies is rare and less feasible in clinical practice. Overall, by providing *in vivo* data, the present study offers supportive knowledge for designing or interpreting clinical investigations regarding the combination of RT with immunotherapy.

In this study, local RT induced dynamic changes in the tumor immune status relevant to PD-1/PD-L1 expression. The altered profiles varied according to the time phases of tumor regression, suggesting the influence of the tumor cell killing and radiation dosing strategy on the tumor immune microenvironment. Further studies are warranted to evaluate the molecular mechanisms underlying these results.

## Data availability statement

The data presented in the study are deposited in the Gene Expression Omnibus (GEO) repository, accession number GSE211916.

## Ethics statement

The animal study was reviewed and approved by Institutional Animal Care and Use Committee of the Korea Institute of Radiological and Medical Sciences (Seoul, Republic of Korea).

## Author contributions

YJL and J-SK conceptualized and supervised this study with funding acquisition. YNY and MHC performed the experiments, formal analysis, and data presentation. WKC and MK assisted in determining the RT technique and dose regimens. YNY and YJL wrote the original draft. YNY, MHC, WKC, MK, J-SK, and YJL reviewed and edited the final manuscript. All authors contributed to the article and approved the submitted version.

## Funding

This work was supported by the National Research Foundation of Korea (NRF) grants funded by the Korea government (Ministry of Science and ICT) (2019R1C1C1007994 and 2021R1C1C1005297 for YL, and 2020M2D9A2094153 and 2020R1A2C1004775 for J-SK).

## Conflict of interest

The authors declare that the research was conducted in the absence of any commercial or financial relationships that could be construed as a potential conflict of interest.

## Publisher’s note

All claims expressed in this article are solely those of the authors and do not necessarily represent those of their affiliated organizations, or those of the publisher, the editors and the reviewers. Any product that may be evaluated in this article, or claim that may be made by its manufacturer, is not guaranteed or endorsed by the publisher.
