# Supplementary material for: Dynamic alterations in PD-1/PD-L1 expression level and immune cell profiles based on radiation response status in mouse tumor model
Source: Front Oncol. 2022 Nov 21;12:989190. doi: 10.3389/fonc.2022.989190 (PMC9720306; doi:10.3389/fonc.2022.989190)
Supplement: Supplementary file 1 [file DataSheet_1.docx]

**
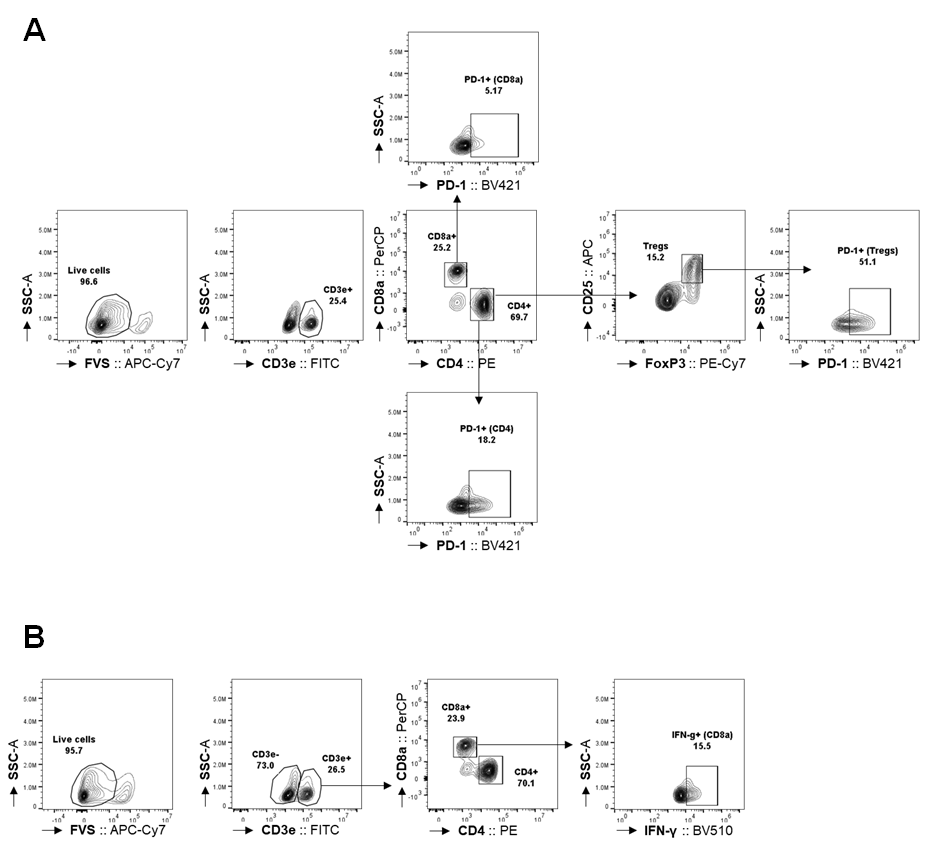
**

**Figure S1.** Gating Strategy for T-cell subsets. (A) Gating strategy of intratumoral and splenic T cells. After single cells were separated by plotting forward-scatter area against side-scatter area and forward-scatter height against width, dead cells were eliminated by gating on FVS-negative plots. All T cell subsets were gated on CD3e^+^ events and then CD8α T cells were distinguished with CD4 T cells. Gated on CD4^+^ events, the brightly stained FoxP3^+^CD25^+^ events were gated as regulatory T cells. (B) Gating strategy of stimulated CD8 α T cells. After gating on FVS^-^CD3e^+^CD8^+^CD4^-^, the brightly stained IFN-γ was analyzed.

**
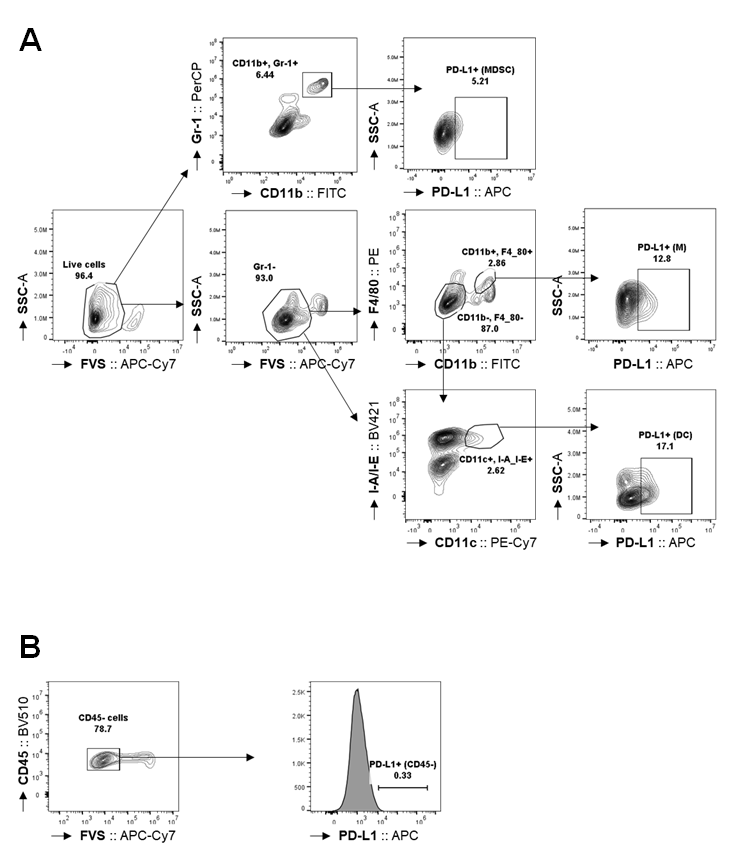
**

**Figure S2.** Gating Strategy for myeloid cells and tumor. (A) Gating strategy of intratumoral myeloid cells. After single cells were separated by plotting forward-scatter against side-scatter area and forward-scatter height against width, dead cells were eliminated by gating on FVS-negative plots. Myeloid-derived suppressor cells (MDSC) were separated by Gr-1^+^CD11b^+^, and macrophages and dendritic cells were gated on Gr-1^-^ events in advance. The brightly stained F4/80^+^CD11b^+^ was distinguished as macrophages (M) and CD11c^+^I-A/I-E^+^ was separated as dendritic cells (DC). (B) Gating strategy of tumor cells. Tumor cells were separated based on CD45^-^ and FVS-negative events.


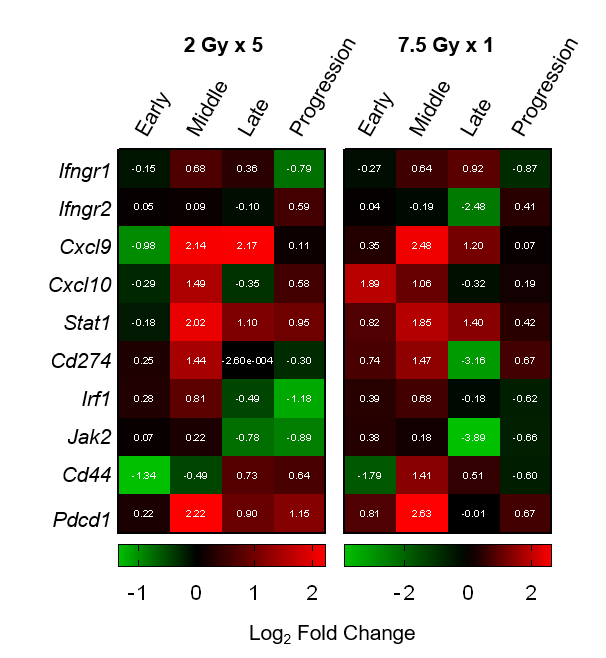


**Figure S3.** Radiation-induced sequential alterations in IFN-γ related genes with respect to immune-stimulatory and suppressive responses. Log_2_ fold change values for each time phase were calculated based on the gene expression at baseline. Abbreviation: IFN, interferon.

**Table S1.** List of monoclonal antibodies used for flow cytometry.


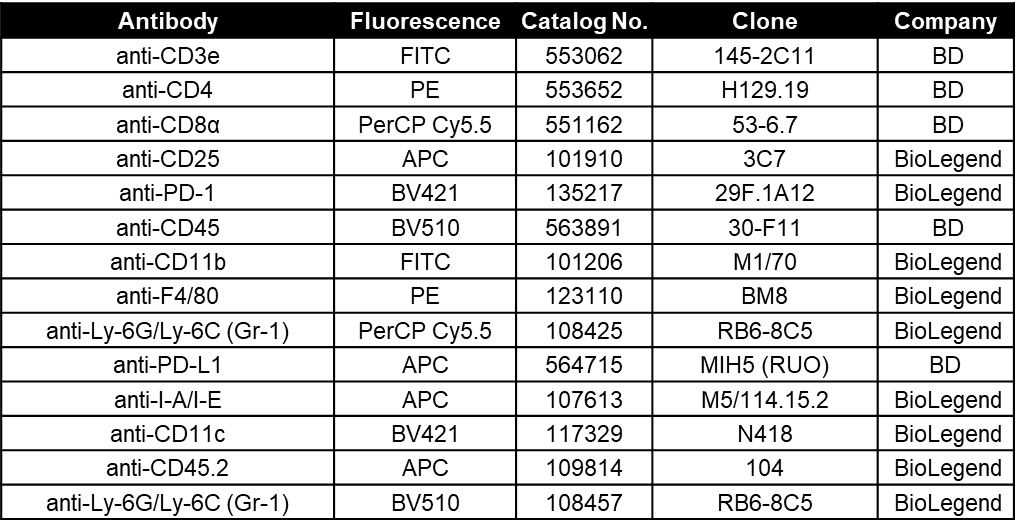


**Table S2.** Gating strategy information of each immune cell.


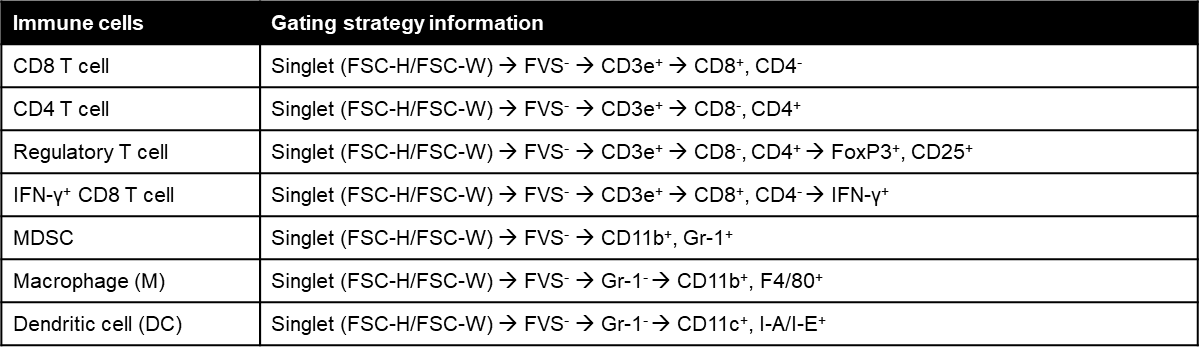


**Table S3.** Monoclonal antibodies used for intracellular staining.


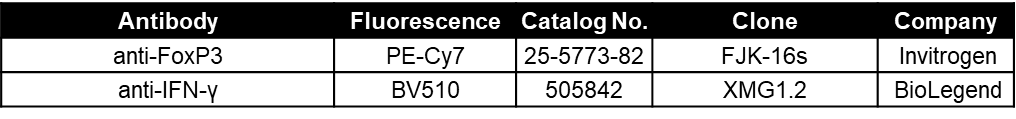


**Table S4.** Results of analysis of variance analyses for comparisons of 0 Gy, 2 Gy × 5, and 7.5 Gy × 1 groups.

| Marker | Type of target cell | Figure No. | Time phase | *P*-value |
| --- | --- | --- | --- | --- |
| PD-L1 | Tumor cells | 2A | Early | 0.305 |
|  |  |  | Middle | 0.001 |
|  |  |  | Late | 0.011 |
|  |  |  | Progression | 0.009 |
|  | MDSCs (intratumoral) | 2D‒E | Middle | 0.007 |
|  |  |  | Progression | 0.032 |
|  | TAMs (intratumoral) | 2F‒H | Middle | 0.040 |
|  |  |  | Progression | 0.020 |
|  | DCs (intratumoral) | 2I‒K | Middle | 0.177 |
|  |  |  | Progression | 0.363 |
| PD-1 | CD8α T cells (intratumoral) | 3A | Early | 0.408 |
|  |  |  | Middle | 0.001 |
|  |  |  | Late | 0.001 |
|  |  |  | Progression | 0.018 |
|  | CD4 T cells (intratumoral) | 3B | Early | 0.306 |
|  |  |  | Middle | 0.287 |
|  |  |  | Late | 0.011 |
|  |  |  | Progression | 0.302 |
|  | Tregs (intratumoral) | 3C | Early | 0.455 |
|  |  |  | Middle | 0.190 |
|  |  |  | Late | 0.003 |
|  |  |  | Progression | 0.682 |
| ‒ | CD8α T cells (splenic) | 4A | Early | 0.261 |
|  |  |  | Middle | 0.265 |
|  |  |  | Late | 0.714 |
|  |  |  | Progression | 0.172 |
|  | CD4 T cells (splenic) | 4B | Early | 0.331 |
|  |  |  | Middle | 0.584 |
|  |  |  | Late | 0.899 |
|  |  |  | Progression | 0.220 |
|  | Tregs (splenic) | 4C | Early | 0.076 |
|  |  |  | Middle | 0.718 |
|  |  |  | Late | 0.133 |
|  |  |  | Progression | 0.732 |
| PD-1 | CD8α T cells (splenic) | 5A | Early | 0.699 |
|  |  |  | Middle | 0.014 |
|  |  |  | Late | 0.012 |
|  |  |  | Progression | 0.029 |
|  | CD4 T cells (splenic) | 5B | Early | 0.920 |
|  |  |  | Middle | 0.666 |
|  |  |  | Late | < 0.001 |
|  |  |  | Progression | 0.083 |
|  | Tregs (splenic) | 5C | Early | 0.846 |
|  |  |  | Middle | 0.893 |
|  |  |  | Late | 0.386 |
|  |  |  | Progression | 0.622 |
| IFN-γ | CD8α T cells (intratumoral) | 6A | Early | 0.967 |
|  |  |  | Middle | 0.010 |
|  |  |  | Late | 0.015 |
|  |  |  | Progression | 0.015 |
